# Postprandial PYY increase by resistant starch supplementation is independent of net portal appearance of short-chain fatty acids in pigs

**DOI:** 10.1371/journal.pone.0185927

**Published:** 2017-10-05

**Authors:** Anne Krog Ingerslev, Shivaprakash Jagalur Mutt, Helle Nygaard Lærke, Mette Skou Hedemann, Peter Kappel Theil, Kirstine Lykke Nielsen, Henry Jørgensen, Karl-Heinz Herzig, Knud Erik Bach Knudsen

**Affiliations:** 1 Department of Animal Science, Aarhus University, Tjele, Denmark; 2 Research Unit of Biomedicine and Biocenter of Oulu, Department of Physiology, University of Oulu, Oulu, Finland; 3 Department of Gastroenterology and Metabolism, Poznan University of Medical Sciences, Poznan, Poland; 4 Medical Research Center (MRC) and University Hospital, Oulu, Finland; National Institute for Agronomic Research, FRANCE

## Abstract

Increased dietary fiber (DF) fermentation and short-chain fatty acid (SCFA) production may stimulate peptide tyrosine-tyrosine (PYY) secretion. In this study, the effects of hindgut SCFA production on postprandial PYY plasma levels were assessed using different experimental diets in a porto-arterial catheterized pig model. The pigs were fed experimental diets varying in source and levels of DF for one week in 3×3 Latin square designs. The DF sources were whole-wheat grain, wheat aleurone, rye aleurone-rich flour, rye flakes, and resistant starch. Postprandial blood samples were collected from the catheters and analyzed for PYY levels and net portal appearance (NPA) of PYY was correlated to NPA of SCFA. No significant effects of diets on NPA of PYY were observed (*P* > 0.05), however, resistant starch supplementation increased postprandial NPA of PYY levels by 37 to 54% compared with rye-based and Western-style control diets (*P* = 0.19). This increase was caused by higher mesenteric artery and portal vein PYY plasma levels (*P* < 0.001) and was independent of SCFA absorption (*P* > 0.05). The PYY levels were higher in response to the second daily meal compared with the first daily meal (*P* < 0.001), but similar among diets (*P* > 0.10). In conclusion, the increased postprandial PYY responses in pigs fed with different levels and sources of DF are not caused by an increased SCFA absorption and suggest that other mechanisms such as neural reflexes and possibly an increased flow of digesta in the small intestine may be involved. The content of DF and SCFA production did not affect PYY levels.

## Introduction

The gastrointestinal tract is a large endocrine organ releasing regulatory peptides in response to nutrient content [1]. Peptide tyrosine-tyrosine (PYY) is a hormone synthesized and released in response to food intake from the endocrine L-cells mainly in the distal part of the gastrointestinal tract, such as ileum and colon, and has several gut functions that contribute to postprandial satiety and decreased food intake [2]. These functions mediate, among others, ileal and colonic breaks to slow gastric emptying and promote digestive activities including regulation of insulin secretion and glucose homeostasis [2, 3].

Dietary fiber (DF) is a nutritional component associated with increased satiety feelings [4]. This is due to properties of adding bulk and producing intraluminal viscosity in the gastrointestinal tract that delay gastric emptying, and inhibit the rate of digestion and absorption of macronutrients [5, 6] with beneficial effects on glycaemia and insulinemia [7]. Furthermore, Karhunen *et al*. [8] showed that in humans a soluble psyllium-fiber enriched meal induced a prolonged increase in PYY concentration. However, Juvonen *et al*. [9] found no short-term PYY effects of oat bran supplemented puddings in healthy subjects, suggesting that the discrepancies might be fiber dependent. Fermentation of DF by microbes in the distal part of the small intestine and colon additionally produces short-chain fatty acid (SCFA), mainly acetate, propionate, and butyrate, that are known to affect PYY release. Previous studies have shown that rectal and ileal infusions of SCFA in rodents, pigs, and humans stimulate PYY release [10–12]. Particularly butyrate has received considerable attention due to its multiple effects on colonic health [13–15]. A recent *in vivo* study in mice showed that oral propionate or butyrate administration increased PYY secretion and reduced feed intake [16]. However, appetite regulation by SCFA is a more long-term regulation since nutrients need to reach the colon before DF fermentation and SCFA absorption can take place.

Based on the importance of SCFA in the release of PYY and its suppressive effect on appetite, we determined the effect of absorbed SCFA on PYY levels by analyzing the plasma PYY concentrations and correlated them with previously studied varying SCFA levels from feed containing different levels of DF. Previously, we studied the effects of resistant starch and rye-based diets on portal absorption of SCFA and insulin sensitivity, demonstrating that a high portal SCFA absorption was associated with lower insulin secretion [17, 18]. In addition, SCFA absorption was higher and insulin levels lower after the second meal in the same day compared to the first meal. Therefore, in this study we investigated if PYY levels are affected by DF supplementations and how these changes correlate with the net portal appearance (NPA) of SCFA. Therefore, plasma samples from two separate and previously conducted studies [17, 18] were analyzed for PYY concentrations. The experimental diets used differed in their fermentative capacity, causing different absorption of SCFA. We hypothesized that an increased intake of DF and hence an augmented SCFA absorption would increase PYY concentrations.

## Materials and methods

### Experimental diets

The experimental diets provided all the nutrients, vitamins and minerals required by growing pigs (S1 and S2 Tables). These diets contained varying amounts of DF to either have high or low effects on colonic fermentation and SCFA absorption. The experimental details and the composition of the diets are summarized in the flowchart (S1 Fig).

The diets consisted of a low-DF Western-style diet (WSD) based on white wheat flour, and two high-DF diets, an arabinoxylan-rich diet (AXD) and a resistant-starch rich diet (RSD) [18] or wheat whole grain (WWG), wheat aleurone-rich flour (WAF) and rye aleurone-rich flour (RAF) [17]. Diets were formulated to be balanced with regard to protein, fat, and metabolizable energy, but to significantly differ in DF content (Table 1). This was successfully achieved in the different diets with the same energy density of the diet (19.24–20.43 MJ), except for the protein content in AXD. This difference was the result of a higher than estimated protein content of the wheat flour incorporated in the RSD and WSD and thus a lower protein content in the AXD [19]. Consumption of AXD resulted in the highest NPA of total SCFA (102 nmol/h), with intermediate levels after RSD consumption (66 mmol/h), and lowest levels in WSD-fed pigs (37 mmol/h, *P*_*Diet*_ = <0.001) [18] (Reprinted with permission). Consumption of the RAF tended to increase the NPA of SCFA (46 mmol/h), compared to intermediate levels in the WAF-fed pigs (39 mmol/h), and the lowest levels in the WWG-fed pigs (33 mmol/h, *P*_*Diet*_ = 0.07) [17] (Reprinted with permission). In addition, a parallel study with intact pigs fed the same experimental diets as in experiment 1 of the present study, Nielsen *et al*. [19] found a higher proportion of digesta residues, particularly as starch degradation products, in the distal small intestine 1.5 hours after feeding in response to the RSD compared to the WSD and AXD diets.

**Table 1:** Chemical composition of the experimental diets and postprandial net portal appearance of total SCFA after consumption of the experimental diets.

	Experimental diets					
	Experiment 1			Experiment 2		
	WSD	AXD	RSD	WWG	WAF	RAF
DM^a^ (g/kg as-fed basis)	915	891	903	654	704	691
**Chemical composition (units/kg DM)**						
Gross energy, MJ	19.68	19.24	20.30	20.35	20.35	20.43
Ash	37	51	34	43	58	41
Protein (N × 6.26), g	207	154	191	173	177	173
Fat, g	152	135	150	137	134	146
Digestible carbohydrates, g	535	442	473	525	484	522
Sugars, g	113	22	3	19	14	17
Starch, g	422	420	470	506	470	505
Non-digestible carbohydrates, g^b^	72	169	183	116	115	122
Resistant starch, g	6	8	113	11	10	18
Total NSP (soluble), g	58 (11)	144 (33)	55 (8)	105 (26)	105 (15)	104 (39)
Fructans, g	0	22	3	3	2	10
AX (soluble)	18 (6)	72 (22)	15 (4)	61 (17)	62 (9)	50 (19)
Klason lignin, g	6	15	13	22	23	26
Total dietary fiber, g^c^	70	167	181	138	138	148
NPA of SCFA (mmol/h)^d^	37	102	66	33	39	46

Diets were analyzed as described in the Materials and Method section. WSD, Western-style diet; AXD, arabinoxylan-rich diet; RSD, resistant starch-rich diet; WWG, whole-wheat grain; WAF, wheat aleurone flour; RAF, rye aleurone flour.

^a^ Abbreviations: DM, dry matter; N, nitrogen; NSP, non-starch polysaccharides.

^b^ Calculated as fructans + resistant starch + total NSP + lignin.

^c^ Calculated as non-digestible carbohydrates + Klason lignin

^d^ The statistical significance of diet was *P*_*Diet*_ < 0.001 in experiment 1, and *P*_*Diet*_ = 0.07 in experiment 2. Reprinted with permission [17, 18].

### Ethics statement

The animal experiments were conducted according to licenses obtained from the Danish Animal Experiments Inspectorate, Ministry of Food, Agriculture and Fisheries, Danish Veterinary and Food Administration. The studies were in compliance with the guidelines concerning animal experiments and care of animals under study according to the Danish Ministry of Justice, Act. 726 of September 9, 1993, and as amended in Act 1306 of November 23, 2007. The health of the animals was monitored throughout the experimental period, and no serious illnesses were observed. All surgery was performed under general anesthesia, and all efforts were made to minimize suffering by providing postsurgical analgesia. After the end of the experiments, the pigs were euthanized with an overdose of pentobarbital, followed by bleeding for postmortem autopsy. Plasma samples were collected from previously conducted animal experiments and evaluated in a novel context [17, 18].

### Animals and experimental design

The pigs were obtained from Department of Animal Science, Aarhus University Foulum, Tjele, Denmark. The animals were surgically equipped with catheters in the portal vein and mesenteric artery [17, 18]. The experimental period lasted for three consecutive weeks and was designed as a repeated 3×3 Latin square design. In each experimental week, the pigs were adapted to the experimental diets for 4–6 days before blood samples were collected on days 7, 14, and 21 as described by others [20, 21].

In each experiment six female Danish landrace × Yorkshire pigs (58.8 (SEM 1.6) kg) (experiment 1) or (56.5 (SEM 1.8) kg) (experiment 2) were included.

#### Experiment 1: Feeding WSD, AXD, and RSD

The WSD supplied 70 g DF per day, and the RSD and AXD supplied 192 and 189 g DF per day, respectively, for a period of 1 week on each diet. Meal portions were weighed from day-to-day and divided into three meals per day, given at 09:00, 14:00, and 19:00 hours supplying 33.3% to mimic a typical human meal pattern. Consecutive blood samples were collected on days 7, 14, and 21 at -15 min (first daily meal fed at 0 min), 15, 30, 45, 60, 90, 120, 180, 240, and 300 min relative to feeding the first daily meal. Plasma SCFA was analyzed at time points 0, 60, 180, and 300 min relative to the first daily meal. Blood samples were collected in Na-heparin vacutainers, and plasma was collected by centrifugation (12 min, 2000*g*, 4°C).

#### Experiment 2: Feeding WWG, WAF, and RAF

The animals received one of the three experimental diets on days 4–7, after having been fed with a washout diet for an initial 3 days of the experimental week. The daily ration supplied 210 g DF per day and was weighed from day-to-day. The daily ration was divided into three meals given at 09:00, 14:00, and 19:00 hours providing 40, 40 and 20% of the daily feed allowance to mimic the diurnal variation in the human intake of DF. Consecutive blood samples were collected at days 7, 14, and 21 at 0 min (first daily meal), 30, 60, 120, 180, 240, 300 min (second daily meal), 330, 360, 420, 480, 540, and 600 min relative to feeding the first daily meal. Blood samples were collected in Na-heparin vacutainers, and plasma was collected by centrifugation (12 min, 2000*g*, 4°C). Plasma samples for arterial and portal SCFA analyses were pooled in 4 pools (pool 1–4) on each sampling day to represent the mean SCFA content in the blood within each pig. One and a half mL arterial and 1.5 mL portal plasma were pooled for arterial and portal SCFA analyses, respectively, from time points 15, 60, and 120 min (pool 1), 180, 240, and 300 min (pool 2), 315, 360, and 420 min (pool 3), and 480, 540, and 600 min (pool 4).

### Analytical methods

Portal venous and arterial plasma samples were analyzed for PYY (total) using the MILLIPLEX MAP Mouse Metabolic Hormone Magnetic Bead Panel, Antibody-Immobilized Magnetic PYY beads having 3–36 amino-acid sequences with 100% specificity to the rat, mouse, canine and porcine species (Millipore, Billerica, MA, USA) with an inter-assay CV of 5.6–6.6% and 5–17% and intra-assay CV of 6.0–8,4% and 4–13% in experiments 1 and 2, respectively. Plasma SCFA was determined as previously described [17, 18].

### Calculations and statistics

Net portal appearance (NPA) was calculated as described by Rerat *et al*. [22]:

$$NPA=PPF\;\times \left(c_{\left(PV\right)}-c_{\left(MA\right)}\right)$$ (1)

where PPF is the portal plasma flow, c_(PV)_ is the concentration in the portal vein, and c_(MA)_ is the concentration in the mesenteric artery. For the correlations between NPA of PYY and SCFA in experiment 2, an average NPA of PYY was calculated using mean PYY concentrations and mean blood flows at the following time intervals after feeding: time points 30, 60, and 120 min (pool 1); 180, 240, and 300 min (pool 2); 330, 360, and 420 min (pool 3); 480, 540, and 600 min (pool 4).

Effects of diet, time, and their two-factor interactions in experiment 1 were analyzed as repeated measurements using the MIXED procedure of Statistical Analysis Software (SAS, version 9.3, SAS Institute Inc., Cary, NC, USA). Plasma variables were analyzed as a linear mixed model

$$Y_{ijkl}=\mu +\alpha_{i}+\beta_{j}+\alpha \beta_{ij}+\gamma_{k}+\delta_{ikl}+\rho_{ijkl}+\varepsilon_{ijkl}$$ (2)

where *Y*_*ijkl*_ is the dependent variable; *μ* is the overall mean; *α*_*i*_ is the effect of diet (*i* = WSD, RSD, AXD); *β*_*j*_ is the time after the first daily meal on day 7 (*j* = -15, 15, 30, 45, 60, 120, 180, 240, and 300 min); and *αβ*_*ij*_ is the interaction term. The three terms *γ*_*k*_ (*k* = pig 1, 2, 3, 4, 5, 6), *δ*_*ikl*_ (*l* = week 1, 2, 3), and *ρ*_*ijkl*_ accounted for repeated measurements being performed on the same pig (*γ*_*k*_) each week (*δ*_*ikl*_) and on the same pig within a sampling period (*ρ*_*ijkl*_), while *ε*_*ijkl*_ describes the residual error component. Fasting concentrations were calculated without effects of time and diet-time interactions. The covariance structure of *ρ*_*ijkl*_ was modelled using the spatial power option, which takes into account the different intervals between repeated measurements. The random effects and residuals are assumed to be normally distributed and independent and their expectations were assumed to be zero.

Effects of the first and second meal in experiment 2 were analyzed as repeated measurements using the MIXED procedure of Statistical Analysis Software (version 9.3, SAS Institute Inc., Cary, NC, USA). Plasma variables were analyzed as a linear mixed model

$$\begin{array}{c} Y_{ijklmn}=\mu +\alpha_{i}+\beta_{j}+\gamma_{k}+\alpha \beta_{ij}+\alpha \gamma_{ik}+\beta \gamma_{jk}+\alpha \beta \gamma_{ijm}+\delta_{l}+\rho_{m}+\vartheta_{ilm}+\tau_{ilmn}\;+\varepsilon_{ijklm} \end{array}$$ (3)

where *Y*_*ijklmn*_ is the dependent variable; *μ* is the overall mean; *α*_*i*_ is the effect of diet (*i* = WWG, WAF, or RAF); *β*_*j*_ is the effect of meal (*j* = 1 or 2), and *γ*_*k*_ is the time after a meal (*k* = 0, 30, 60, 120, 180, 240 and 300 min), and *δ*_*l*_ is the effect of week (*l* = 1, 2, or 3). The four terms *αβ*_*ij*_, *αγ*_*ik*_, *βγ*_*jk*_, and *αβγ*_*ijk*_ accounted for the interactions between the main effects. The covariance structure *ρ*_*m*_ (*m* = pig) accounted for repeated measurements being performed on the same pig fed different diets each week (*ϑ*_*ilm*_) and on the same pig within a sampling period (*τ*_*ilmn*_), and was modelled using the spatial power option, which takes into account the adverse time intervals between repeated measurements. The *ε*_*ijklm*_ is the residual error component under the assumptions that the random effects and residuals are normally distributed and independent, and their expectations were assumed to be zero.

The hormone concentrations were transformed to ln(x) before statistical analysis to obtain variance homogeneity and presented as least square means with 95% confidence intervals. The NPA was calculated on original scale and presented as least square means with standard error (SE). Pearson correlations were evaluated using the PROC CORR procedure of SAS (version 9.3, SAS Institute Inc., Cary, NC, USA). The significance level was set at *P* < 0.05 while *P* ≤ 0.10 was reported being a tendency.

## Results

### Net portal appearance of PYY and plasma concentrations

#### Experiment 1: Effect of WSD, AXD, and RSD

**Fig 1:**
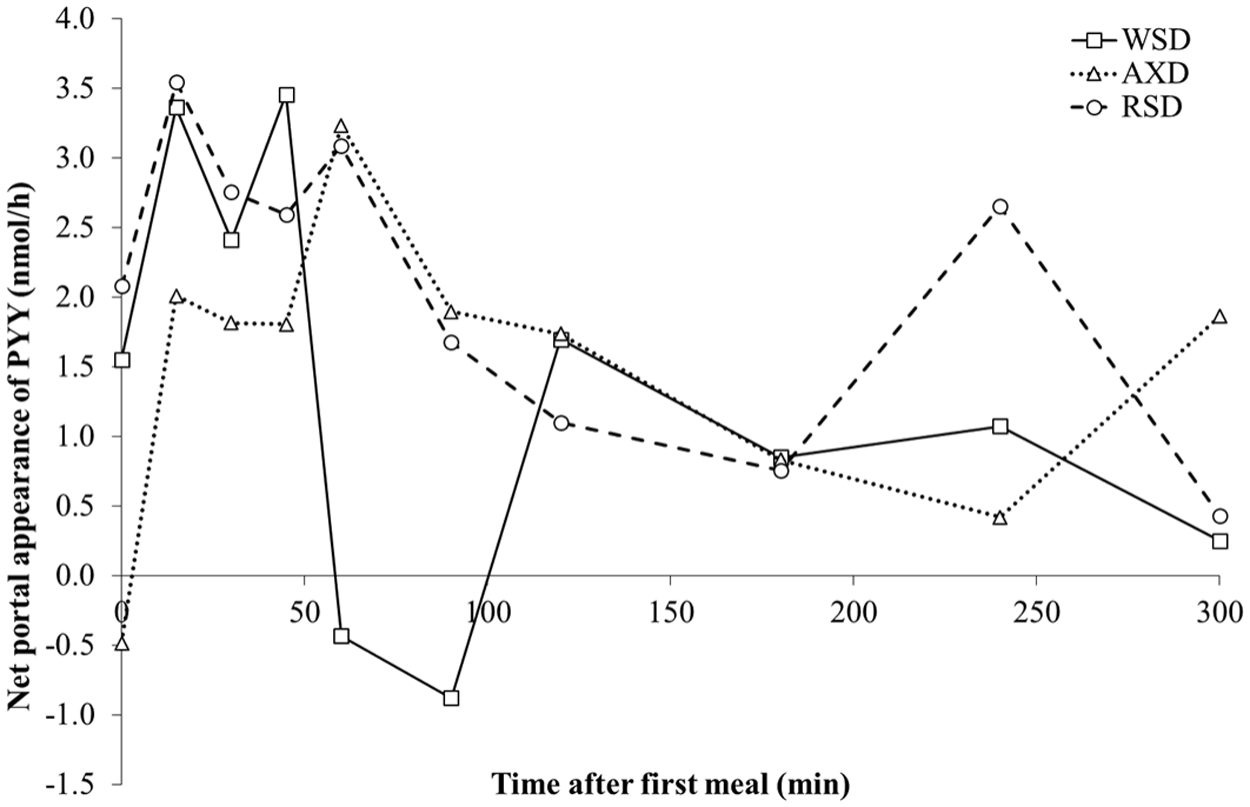
Net portal appearance of PYY in pigs fed WSD, AXD, and RSD. Net portal appearance of PYY following the first daily meal (0 min). Values are means, n = 6; *P*_*Diet*_ = 0.19, *P*_*Time*_ = 0.03, *P*_*Diet×Time*_ = 0.14). The solid lines represent means from pigs fed the Western-style diet (WSD); dotted lines represent means from pigs fed the arabinoxylan-rich whole-grain diet (AXD); the dashed line represent means from pigs fed the resistant starch-rich diet (RSD).

The 5 h postprandial NPA of PYY was 37–54% higher in response to RSD consumption compared to AXD and WSD intake, although this difference was not statistically significant (*P*_*Diet*_ = 0.19; Table 2). Pigs fed RSD secreted 2.07 nmol/h, compared to 1.34 nmol/h and 1.51 nmol/h in WSD and AXD fed pigs, respectively (Table 2). The PYY secretion increased postprandially on all three diets (*P*_*Time*_ = 0.03; Fig 1) followed by a decrease. The NPA of PYY peaked at 3.4–3.5 nmol/h 15 and 45 min after feeding in RSD and WSD-fed pigs, respectively, and at 3.2 nmol/h after 60 min in response to AXD intake (Fig 1). Fasting and postprandial mesenteric artery and portal vein PYY concentrations were higher in response to RSD compared to WSD and AXD (*P*_*Diet*_ < 0.01; Table 2; S2 Fig). All experimental diets resulted in a rapid increase in mesenteric arterial and portal vein PYY concentrations in response to a meal, followed by a gradual decrease over the next 4 hours (*P*_*Time*_ < 0.001; S2 Fig). Arterial PYY concentrations tended to be higher in response to RSD compared to WSD and AXD throughout the sampling period (*P*_*Diet×Time*_ = 0.053). Mesenteric arterial PYY concentrations peaked at 220 and 183 pM 60 min after feeding RSD and WSD, respectively, as compared with 156 pM 45 min after feeding AXD. Portal vein PYY concentrations in response to RSD and AXD peaked 60 min after feeding at 250 and 169 pM, respectively, compared to 203 pM 30 min after feeding WSD.

**Table 2:** Postprandial net portal PYY appearance and mesenteric artery and portal vein PYY concentrations of pigs fed WSD, AXD, and RSD.

		Experimental diets			SE	*P*-values		
		WSD	AXD	RSD		Diet	Time	Diet×Time
Net portal appearance (nmol/h)		1.34	1.51	2.07	0.38	0.19	0.03	0.14
Arterial PYY (pM)	Fasting	105^b^	120^b^	146^a^	14	0.004	-	-
	Postprandial	140 [114; 172]^b^	135 [110; 165]^b^	182 [148; 223]^a^	-	< 0.001	< 0.001	0.053
Portal PYY (pM)	Fasting	124^b^	113^b^	173^a^	12	0.001	-	-
	Postprandial	157 [130; 189]^b^	147 [122; 177]^b^	203 [169; 245]^a^	-	< 0.001	< 0.001	0.55

Postprandial net portal appearance of PYY and PYY concentrations in the mesenteric artery and portal vein of pigs fed the experimental diets in response to first the daily meal. Fasting concentrations and net portal appearance are means of time points relative to the first daily feeding (-15, 15, 30, 45, 60, 120, 180, 240, and 300 min.) with standard error (SE), and postprandial concentrations are least square means [95% cis], n = 6.

^a,b^Means within a row without common superscript differ (*P* < 0.05).

WSD, Western-style diet; AXD, arabinoxylan-rich whole-grain diet; RSD, resistant starch-rich diet.

#### Experiment 2: Effect of WWG, WAF, and RAF

**Fig 2:**
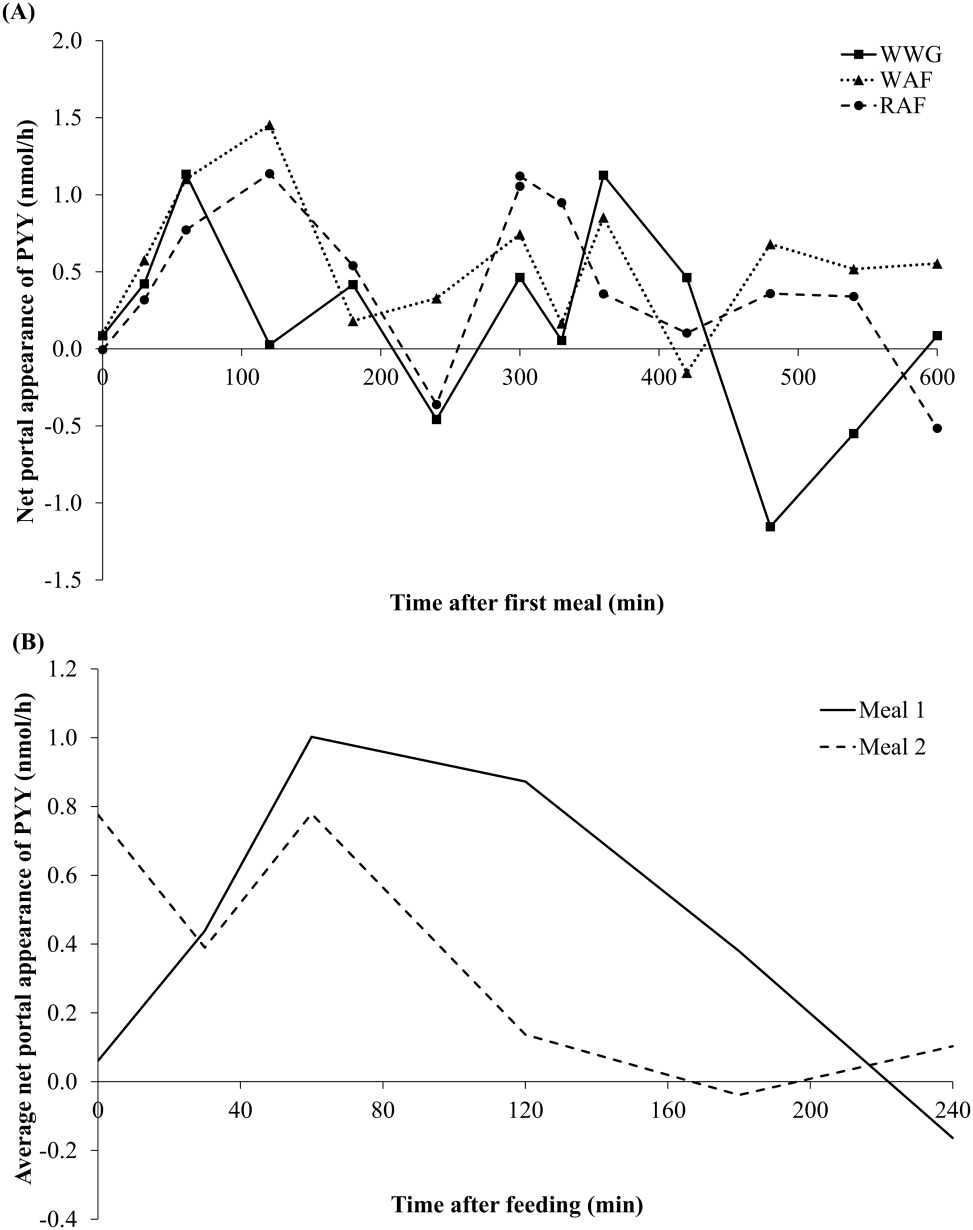
Net portal appearance of PYY following the first (0 min) and second (300 min) daily meals in pigs fed WWG, WAF, and RAF. (A) Net portal appearance of PYY after the first (0 min) and second (300 min) daily meal (*P*_*Diet*_ = 0.21, *P*_*Meal*_ = 0.33, *P*_*Time*_ = 0.11, *P*_*Meal×Time*_ = 0.87, *P*_*Diet×Meal*_ = 0.96, *P*_*Diet×Time*_ = 0.87, *P*_*Diet×Time×Meal*_ = 0.34). Values are means, n = 6. The solid lines represent means from pigs fed the whole-wheat grain diet (WWG); dotted lines represent means from pigs fed the wheat aleurone flour diet (WAF); dashed lines represent means from pigs fed the rye aleurone flour diet (RAF). (B) Average net portal appearance of PYY for the three experimental diets following the first (solid line) and second daily meal (dashed line).

Overall, the NPA of PYY was not affected by diet, meal, time, or any interactions hereof (*P* > 0.05; Table 3). The NPA of PYY displayed an inclination towards a postprandial increase after the first daily meal (Fig 2A), although not statistically significant (*P*_*Diet×Time×Meal*_ = 0.34; Table 3). The NPA peaked at 1.1–1.5 nmol/h 60–120 min after feeding, followed by a decrease. The second daily meal caused a secondary increase, although less prominent as compared to the first response (Fig 2A). The average NPA of PYY for all three diets suggested a more distinct postprandial increase followed by a decrease in response to the first meal compared to the second meal which appeared more fluctuating (Fig 2B), although not statistically significant (*P*_*Meal*_ = 0.33). Postprandial PYY concentrations in the mesenteric artery and portal vein were not different between the three experimental diets (*P*_*Diet*_ > 0.1; Table 3). Mesenteric artery and portal vein PYY concentrations at day 7 following the first daily meal showed a fast increase the first 60 min postprandial after which a plateau phase was reached until the second daily meal 5 hours/300 min later (S3 Fig). The second daily meal caused a drop in PYY concentrations at 330 min with a subsequent rise at 360 min (*P*_*Meal×Time*_ < 0.001). Plasma PYY concentrations reached a higher postprandial plateau after the second daily meal (420 min) as compared with the plateau after the first daily meal (120 min). Overall, PYY concentrations were higher after the second meal compared with the first meal (*P*_*Meal*_ > 0.001; Table 3). Postprandial mesenteric arterial concentrations increased by 12–34% (from 129–137 pM to 154–163 pM) after the first daily meal to the second meal, and portal vein concentrations increased by 9–25% (from 135–142 pM to 155–169 pM) after the first to second meal.

**Table 3:** Postprandial net portal appearance of PYY and PYY concentrations in the mesenteric artery and portal vein of pigs fed of pigs fed WWG, WAF, and RAF^1^.

	Experimental diets			*P*-values						
	WWG	WAF	RAF	Diet	Time	Meal	Meal×Time	Diet×Meal	Diet×Time	Diet×Time×Meal
*Net portal appearance (nmol/h)*	*184*	*561*	*440*	0.21	0.11	0.33	0.17	0.96	0.87	0.34
1. meal	298	641	494							
2. meal	70	480	387							
*Arterial PYY (pM)*										
1. meal	129 [107;154]	130 [108;157]	137 [114;164]	0.87	< 0.001	< 0.001	< 0.001	0.99	0.97	0.97
2. meal	154 [128;184]	155 [129;187]	163 [136;196]							
*Portal PYY (pM)*										
1. meal	135 [115;157]	136 [116;160]	142 [122;167]	0.76	< 0.001	< 0.001	< 0.001	0.89	0.91	0.84
2. meal	155 [133;181]	159 [135;187]	169 [144;198]							

^1^ Postprandial net portal appearance and concentrations are means of time points relative to the first daily feeding (meal 1; 0, 30, 60, 120, 180, 240, 300 min (meal 2) 330, 360, 420, 480, 540, and 600 min), n = 6. Arterial and portal concentrations are given with 95% cis, and net portal appearance with a standard error of 157. WWG, whole-wheat grain; WAF, wheat aleurone flour; RAF, rye aleurone flour.

### Correlations between SCFA absorption and PYY secretion

**Fig 3:**
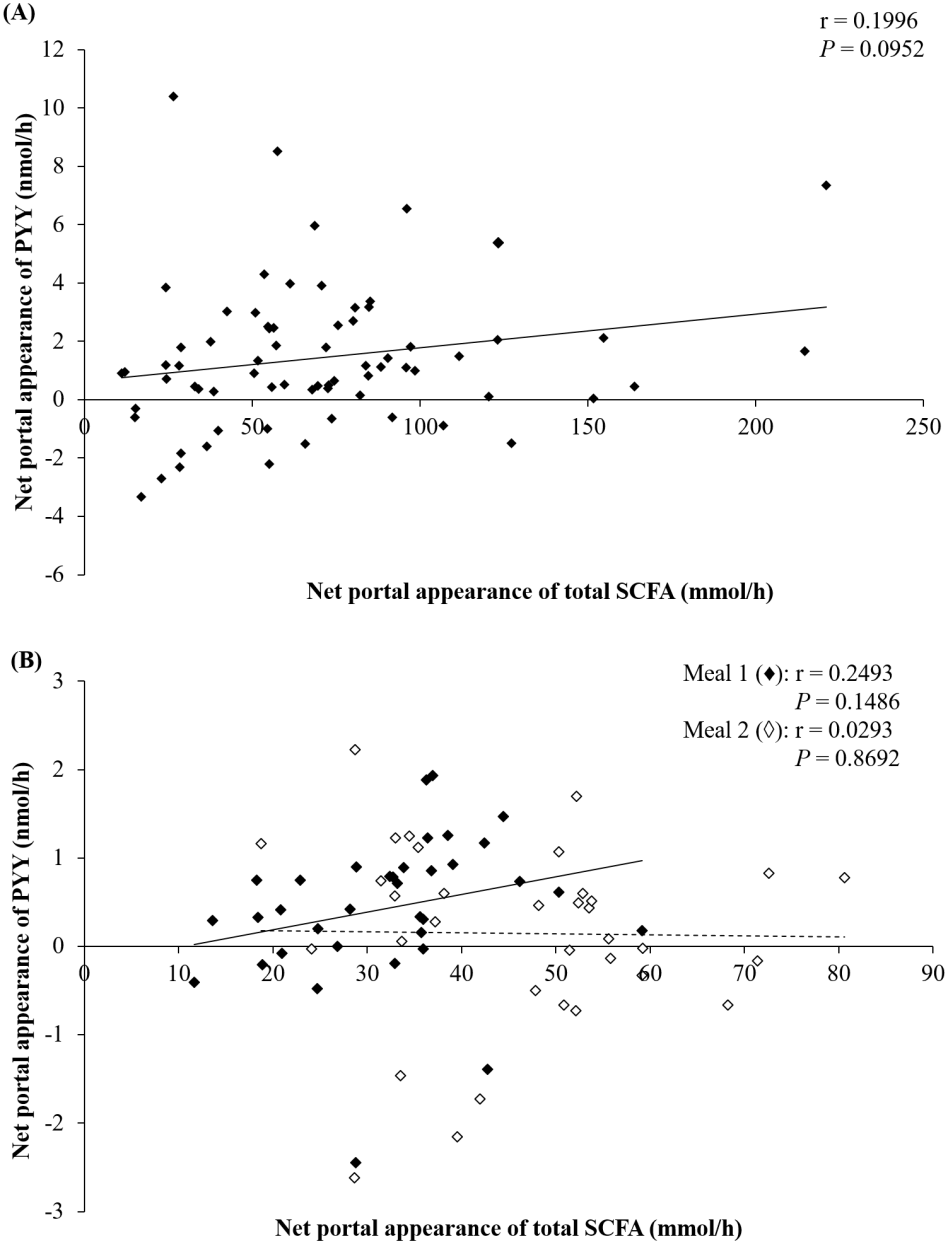
Correlations between net portal appearance of PYY and NPA of total SCFA in pigs fed WSD, AXD, and RSD. (A) Correlation between net portal appearance of PYY and SCFA for each pig after consumption of WSD, AXD, or RSD, n = 6. Each point represents a correlation between net portal appearance of PYY and SCFA for a given pig on a specific diet (time points 0, 60, 180, and 300 min relative to first daily meal). (B) Correlation with first (♦) and second (◊) meals after consumption of WWG, WAF, or RAF, n = 6. Each point represents the average net portal appearance of PYY (pools 1–4 as described in the Materials and Methods) correlated to the net portal appearance of SCFA (pools 1–4) for each pig on each experimental diet. Inserts show the Pearson’s r correlation and the corresponding *P*-value. Solid line represent means for meal 1, and the dashed line represent means for meal 2.

Pearson correlations were used to assess whether there were correlations between NPA of PYY and NPA of total SCFA (Fig 3) for the individual time points. No significant correlations (*P* > 0.05) were found between NPA of PYY and NPA of total or individual SCFA (acetate, propionate, butyrate; *P* > 0.05), as shown in Fig 3 and S3 Table. A Pearson correlation comparing the NPA of SCFA against the NPA of PYY for RSD and WSD fed pigs from experiment 1 specifically, did not reveal significant correlations (*P* = 0.14; S4 Fig).

## Discussion

### Effects of SCFA on PYY levels and secretion

In the present study, we found no significant correlation between SCFA absorption to the portal vein and PYY secretion in pigs in spite of a significant higher SCFA absorption obtained with AXD and RSD in comparison with WSD in experiment 1. Consumption of RSD resulted in 35% and 38% higher arterial and portal vein PYY concentrations, respectively, compared to PYY levels found in AXD fed pigs. In addition, we found similar PYY levels in portal and arterial plasma, indicating a low hepatic clearance of PYY. Our findings are in accordance with previous observations using *in situ* perfusion of rat liver [23], demonstrating that from portal infusion of up to 500 pM, only up to 10% was extracted by the liver. Increasing the intake of fermentable carbohydrates was previously linked to increased colonic SCFA production and increased endogenous PYY secretions in murine models [24, 25]. The DF level in the RSD diet was increased using high-amylose maize and raw potato starch, which was shown to increase PYY concentrations in rodents [25, 26]. In addition, a 3-day human intervention study by Nilsson *et al*. [27] found that ingestion of barley-kernel based bread increased serum SCFA and significantly elevated the plasma PYY levels. The same results were reported by Sandberg *et al*. [28] who demonstrated that whole grain rye kernel bread given as a late evening meal increased both fasting and postprandial plasma concentrations of PYY, glucagon-like peptide 1 (GLP-1), and fasting SCFA the following morning compared to white wheat flour based bread. Brown *et al*. [29] suggested that the PYY increase is mediated by increased colonic SCFA, activating the G-protein-coupled free fatty acid receptors FFAR3 and FFAR2. In the present study, however, postprandial PYY concentrations did not correlate with SCFA levels, as total and individual SCFA concentrations and absorption were lowest in response to WSD, intermediate with RSD, and highest when AXD was fed [18]. We previously found that the changes in SCFA absorption correlated to changes in the fecal microbial composition, particularly in response to the AXD, while the microbial composition did not differ significantly in the RSD compared to either the WSD or the AXD diets [19]. Similarly, despite comparable levels of DF in the WWG, WAF, and RAF based diets, SCFA plasma concentration and absorption were lowest in pigs fed WWG, intermediate in WAF, and highest in response to the rye-based RAF diets [17]. In line with this, we previously found no correlation between the increased SCFA production and colonic FFAR2/FFAR3 gene expression, but discovered an inverse correlation between FFAR3 and FFAR2 gene expression and cecal SCFA/butyrate pool sizes [30]. This apparent discrepancy between increased PYY secretion and lack of effect on FFAR3 gene expression was also recently reported in rats [31]. Adam *et al*. [31] suggested that an increased secretory capacity or an increased number of colonic L-cells was responsible for the increased PYY secretion [31]. Indeed, others have reported increased L-cell number and differentiation in rodents in response to standard diets supplemented with fermentable carbohydrates (oligofructose and fructo-oligosaccharides) [32, 33]. The results presented here thus support previous findings suggesting that increased levels of circulating PYY may be a result of a FFAR3/FFAR2 independent stimulation [16].

The *in vivo* effects of DF and SCFA supplementation on PYY secretion have generated ambiguous results and appear to be related to species differences and animal models used. Production and secretion of PYY are commonly considered to be mediated by L-cells in the distal part of the small intestine and the colon, along with GLP-1 [34]. Recent studies in mice and rats have shown a differential expression of these hormones in the different segments of the intestine with expression of GLP-1 secretion from L-cells dominating in the distal small intestine while both GLP-1 and PYY are expressed in the colon [35–37]. The anatomical distribution of PYY secreting L-cells along the intestinal tract differs between mice, pigs, and humans, especially in the proximal intestine [38, 39]. Interestingly, Cho *et al*. [37] showed that the small intestinal-colon gradient in the expression of PYY was less pronounced in pigs compared to mice, showing that significant inter-species differences exist which may account for the observed differences in PYY responses between rodent and porcine models, and thus possibly humans. Indeed, several rodent studies have shown increased PYY levels after DF consumption [25, 40–42], whereas porcine studies are limited and have produced equivocal results so far [11, 43, 44].

### Effects of digesta residues on PYY absorption

The release of PYY into circulation occurs in response to food intake, and reaches plateau levels within 1–2 hours postprandially followed by a plateau for up to 5 hours in healthy human subjects [45]. This initial release occurs before food enters the distal parts of the gut and it has therefore been suggested that the initial release is neurally mediated in dogs and pigs [46, 47]. The continuous PYY production after an initial increase in plasma PYY levels suggests, however, that the PYY production is related to the presence of digesta residues in the distal small intestine and proximal large intestine acting on ileal and colonic endocrine L-cells. Furthermore, the observation described in the parallel study by Nielsen *et al*. [19], showing a higher proportion of small intestinal digesta residues and starch degradation products present in the small intestines 1.5 hours after feeding in response to the RSD compared to the WSD and AXD, indicate that the effects on postprandial PYY responses reported here are primarily due to neural regulation and ileal digesta residues, rather than SCFA production and absorption. This assumption is supported by the results from experiment 2 where the average second meal PYY increments were significantly higher compared to the first meal. In addition, a parallel study to experiment 2 using ileal cannulated pigs showed an increased flow of digesta in response to the first daily meal compared to the second [48], suggesting that after an overnight fast lasting 14 hours, the small intestine is relatively empty because the small previous evening meal (only 20% of the daily load), has passed through the gastrointestinal tract. Thus, the higher PYY concentrations in response to the second meal could be a result of a cumulative effect of food present in the small intestine.

In conclusion, the results of the present study suggest that in pigs fed diets varying in DF with comparable digestibility in the small intestine and variable SCFA and butyrate NPA total DF intake and SCFA production *per se* were not responsible for the increased PYY secretion. The increased flow of intestinal digesta residues in combination with neural regulation may be responsible for the increased PYY response in pigs.

## Supporting information

S1 Fig Flowchart of experimental design and diets. Experimental design and diets. Flowchart showing the example of repeated 3×3 Latin square design in experiments. Six pigs completed each experiment. WSD, Western-style diet; AXD, arabinoxylan-rich diet; RSD, resistant starch-rich diet; WFL, white wheat flour (washout diet); WWG, whole-wheat grain; WAF, wheat aleurone flour; RAF, rye aleurone flour.
(TIF)

S2 Fig PYY concentrations following the first daily in pigs fed WSD, AXD, and RSD. PYY concentrations following the first daily meal (0 min) in pigs fed WSD, AXD, and RSD. A) Mesenteric artery PYY concentrations (*P*_*Diet*_ = < 0.001, *P*_*Time*_ < 0.001, *P*_*Diet×Time*_ = 0.0531). B) Portal vein PYY concentrations (*P*_*Diet*_ = < 0.001, *P*_*Time*_ < 0.001, *P*_Diet×Time_ = 0.55). Data was ln-transformed before statistical analysis to obtain variance homogeneity, and back transformed to original scale after statistical analyses. Values are means, n = 6. WSD, Western-style diet; AXD, arabinoxylan-rich whole-grain diet; RSD, resistant starch-rich diet.
(TIFF)

S3 Fig PYY concentrations following the first daily meal in pigs fed WWG, WAF, and RAF. PYY concentrations following the first daily meal (0 min) in pigs fed WWG, WAF, and RAF. Data was ln-transformed before statistical analysis to obtain variance homogeneity, and back transformed to original scale after statistical analyses. A) Mesenteric arterial PYY concentrations at day 7 following the first daily meal (0 min) and second daily meal (300 min). Values are means, n = 6. *P*_*Diet*_ = 0.87, *P*_*Time*_ < 0.001, *P*_*Meal*_ < 0.001, *P*_*Meal×Time*_ < 0.001, *P*_*Diet×Meal*_ = 0.99, *P*_*Diet×Time*_ = 0.97, *P*_*Diet×Time×Meal*_ = 0.97. B) Portal vein PYY concentrations at day 7 following the first daily meal (0 min) and second daily meal (300 min). *P*_*Diet*_ = 0.76, *P*_*Time*_ < 0.001, *P*_*Meal*_ < 0.001, *P*_*Meal×Time*_ < 0.001, *P*_*Diet×Meal*_ = 0.89, *P*_*Diet×Time*_ = 0.91, *P*_*Diet×Time×Meal*_ = 0.84. WWG, whole-wheat grain; WAF, wheat aleurone flour; RAF, rye aleurone flour.
(TIFF)

S4 Fig Correlations between net portal appearance of PYY and net portal appearance of total SCFA in pigs fed the RSD and WSD diets. Correlations from experiment 1 between net portal appearance of PYY and net portal appearance of total SCFA in pigs fed the RSD and WSD diets, n = 6. Each point represents a correlation between net portal appearance of PYY and SCFA for a given pig on a specific diet (time points 0, 60, 180, and 300 min relative to first daily meal).
(TIF)

S1 Table Ingredients list of experimental diets used in experiment 1. (DOCX)

S2 Table Ingredients list of experimental diets used in experiment 2. (DOCX)

S3 Table Correlations between net portal appearance of PYY and total or individual short-chain fatty acids in experiments 1 and 2. Correlations between net portal appearance of PYY (nmol/h) and total or individual short-chain fatty acids (SCFA; mmol/h) in experiments 1 and 2.
(DOCX)
