# Supplementary figures and images for: Postprandial PYY increase by resistant starch supplementation is independent of net portal appearance of short-chain fatty acids in pigs
Source: PLoS One. 2017 Oct 5;12(10):e0185927. doi: 10.1371/journal.pone.0185927 (PMC5628905; doi:10.1371/journal.pone.0185927)

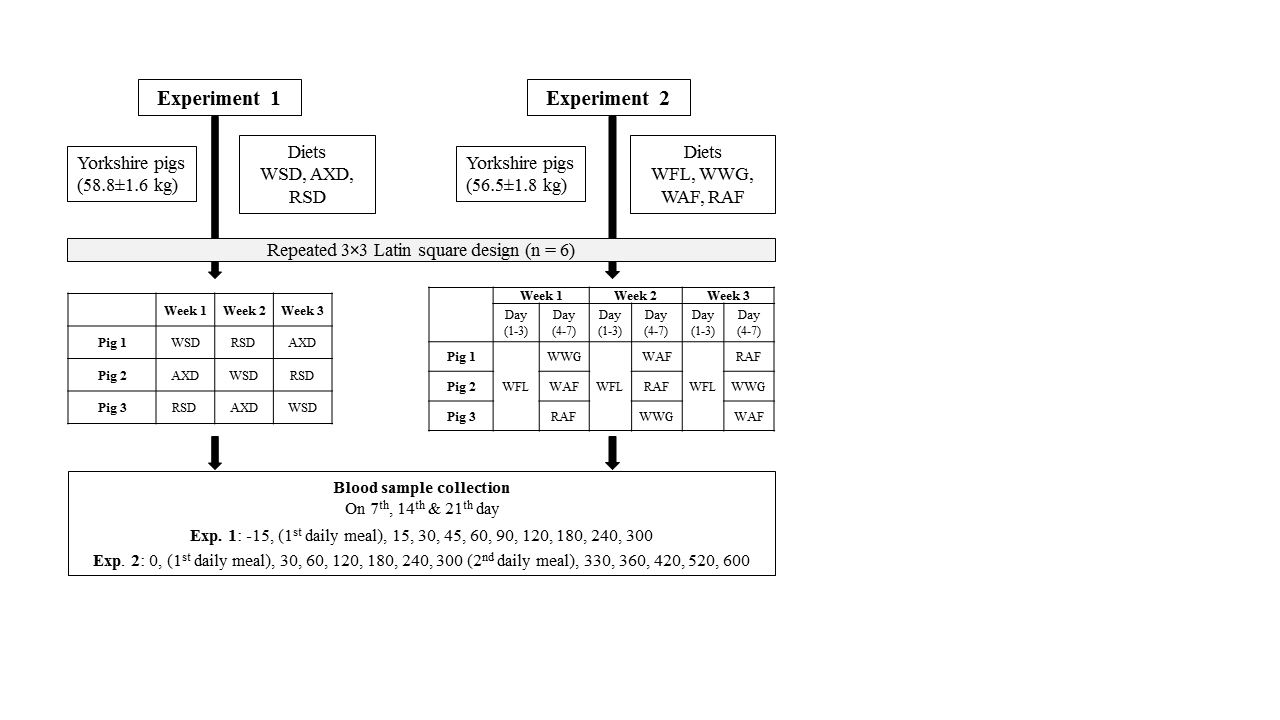

Supplement: S1 Fig — Experimental design and diets. Flowchart showing the example of repeated 3×3 Latin square design in experiments. Six pigs completed each experiment. WSD, Western-style diet; AXD, arabinoxylan-rich diet; RSD, resistant starch-rich diet; WFL, white wheat flour (washout diet); WWG, whole-wheat grain; WAF, wheat aleurone flour; RAF, rye aleurone flour. (TIF) [file pone.0185927.s001.tif]

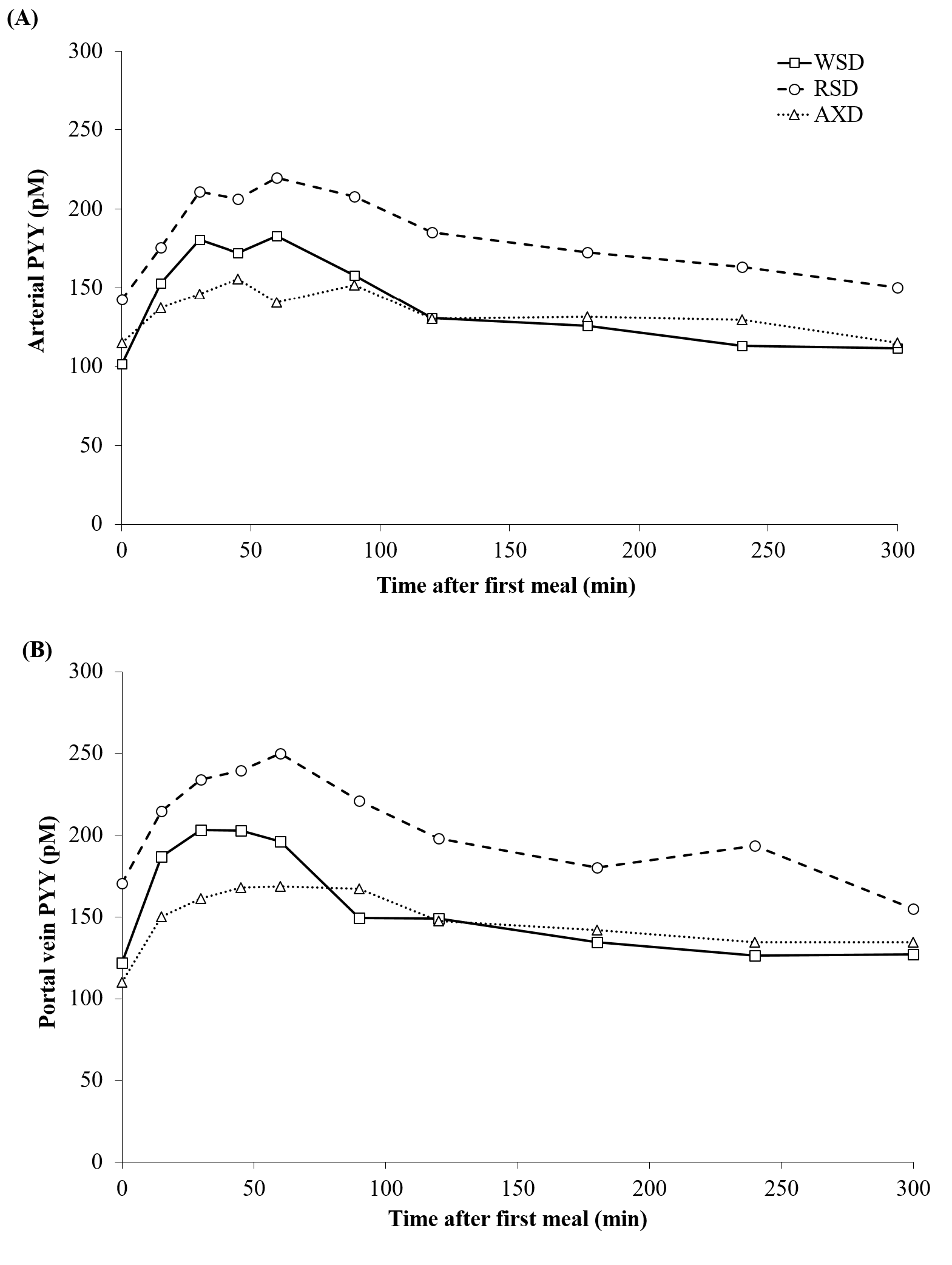

Supplement: S2 Fig — PYY concentrations following the first daily meal (0 min) in pigs fed WSD, AXD, and RSD. A) Mesenteric artery PYY concentrations (PDiet = < 0.001, PTime < 0.001, PDiet×Time = 0.0531). B) Portal vein PYY concentrations (PDiet = < 0.001, PTime < 0.001, PDiet×Time = 0.55). Data was ln-transformed before statistical analysis to obtain variance homogeneity, and back transformed to original scale after statistical analyses. Values are means, n = 6. WSD, Western-style diet; AXD, arabinoxylan-rich whole-grain diet; RSD, resistant starch-rich diet. (TIFF) [file pone.0185927.s002.tiff]

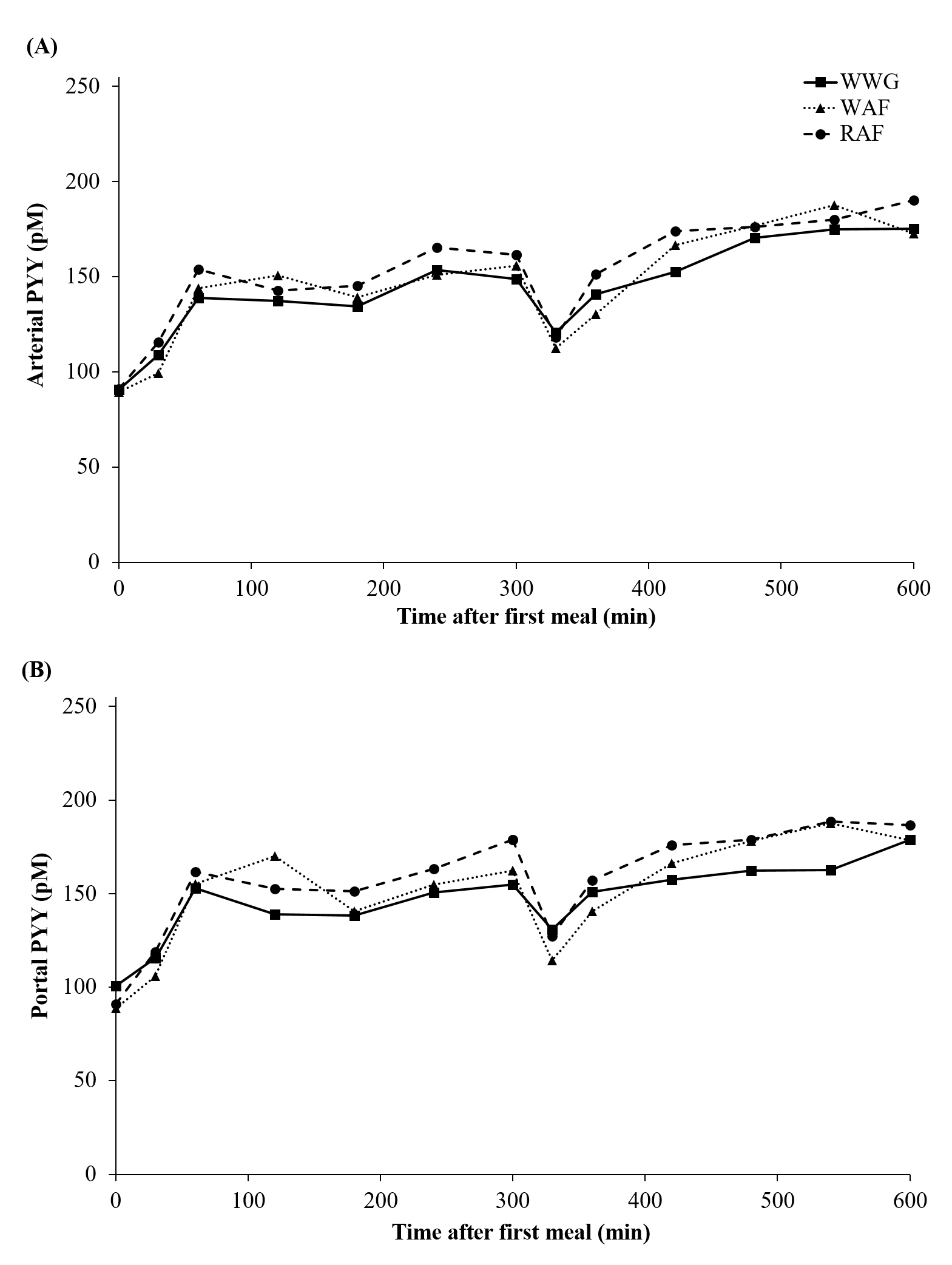

Supplement: S3 Fig — PYY concentrations following the first daily meal (0 min) in pigs fed WWG, WAF, and RAF. Data was ln-transformed before statistical analysis to obtain variance homogeneity, and back transformed to original scale after statistical analyses. A) Mesenteric arterial PYY concentrations at day 7 following the first daily meal (0 min) and second daily meal (300 min). Values are means, n = 6. PDiet = 0.87, PTime < 0.001, PMeal < 0.001, PMeal×Time < 0.001, PDiet×Meal = 0.99, PDiet×Time = 0.97, PDiet×Time×Meal = 0.97. B) Portal vein PYY concentrations at day 7 following the first daily meal (0 min) and second daily meal (300 min). PDiet = 0.76, PTime < 0.001, PMeal < 0.001, PMeal×Time < 0.001, PDiet×Meal = 0.89, PDiet×Time = 0.91, PDiet×Time×Meal = 0.84. WWG, whole-wheat grain; WAF, wheat aleurone flour; RAF, rye aleurone flour. (TIFF) [file pone.0185927.s003.tiff]

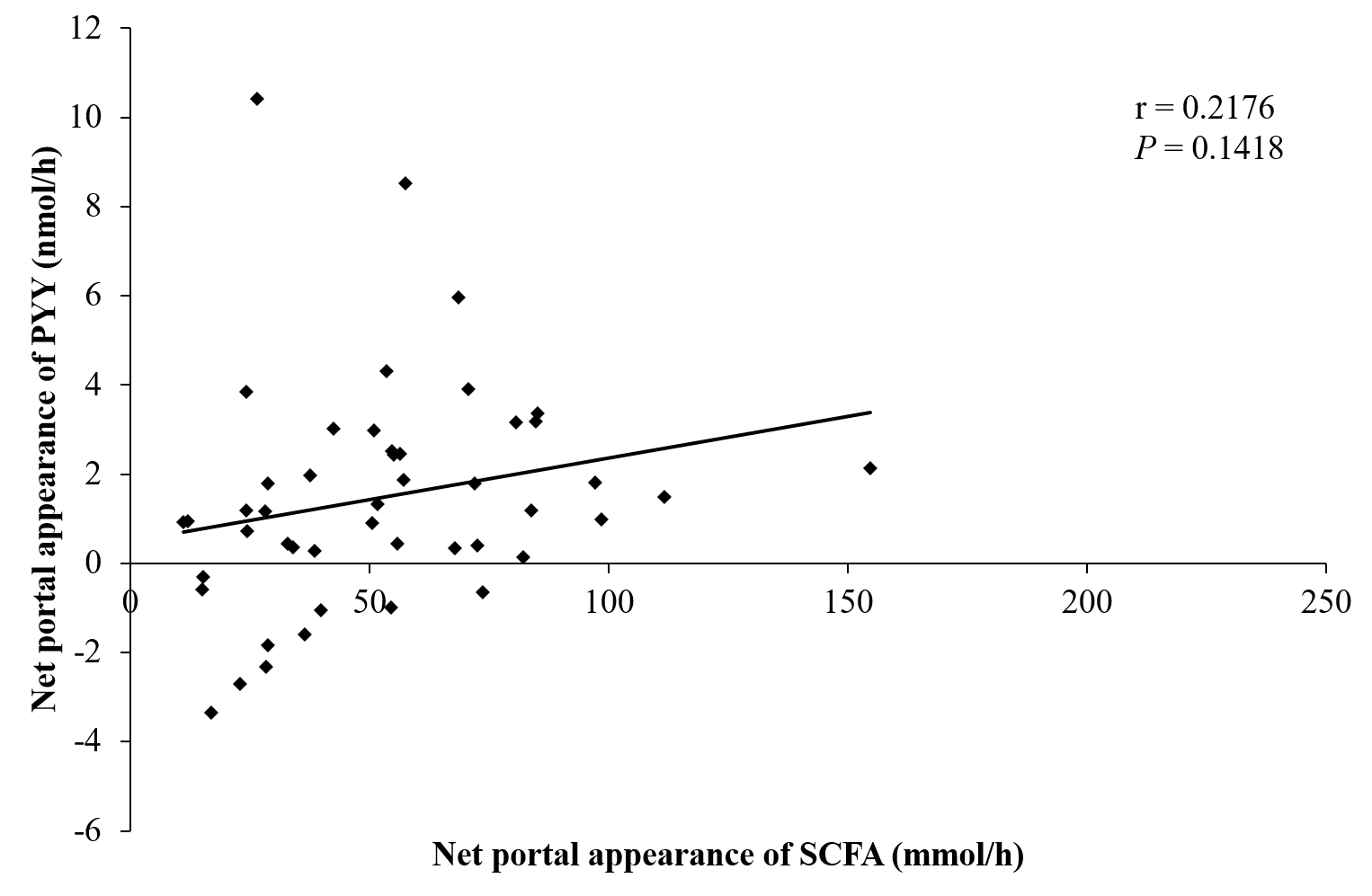

Supplement: S4 Fig — Correlations from experiment 1 between net portal appearance of PYY and net portal appearance of total SCFA in pigs fed the RSD and WSD diets, n = 6. Each point represents a correlation between net portal appearance of PYY and SCFA for a given pig on a specific diet (time points 0, 60, 180, and 300 min relative to first daily meal). (TIF) [file pone.0185927.s004.tif]
