# Supplementary material for: Postprandial PYY increase by resistant starch supplementation is independent of net portal appearance of short-chain fatty acids in pigs
Source: PLoS One. 2017 Oct 5;12(10):e0185927. doi: 10.1371/journal.pone.0185927 (PMC5628905; doi:10.1371/journal.pone.0185927)
Supplement: S1 Table — (DOCX) [file pone.0185927.s005.docx]

**Online Supporting Material**

### Supplemental Table 1: Ingredients list of experimental diets used in experiment 1.

|  | **Experiment 1** | | |
| --- | --- | --- | --- |
|  | WSD | AXD | RSD |
| Ingredients, (g/kg, as-fed basis) * |  |  |  |
| White wheat flour | 568 | - | 455 |
| Rye flakes | - | 655 | - |
| Enzyme treated wheat bran | - | 80 | - |
| Raw potato starch | - | - | 56 |
| HiMaize® (60% resistant starch) | - | - | 168 |
| Lard | 96 | 80 | 89 |
| Soy bean oil | 32 | 27 | 30 |
| Sugar | 100 | 0 | 0 |
| Lacprodan 87 (Whey protein) ^¶^ | 131 | 85 | 129 |
| Vitacel WF600 (73% cellulose)^†^ | 40 | 40 | 40 |
| Vitamin-mineral mixture^‡^ | 30 | 30 | 30 |
| Chromic oxide | 3 | 3 | 3 |

WSD, Western-style diet; AXD, arabinoxylan-rich whole-grain diet; RSD, resistant starch-rich diet.

® a registered trademark of Ingredion Incorporated, Bridgewater, NJ, USA

* Formulated to supply 53% E from carbohydrates, 17% E from protein, and 30% E from fat

¶ Arla Foods Ingredients amba, Viby J, Denmark

† J. Rettenmaier and Söhne GmbH, Rosenberg, Germany

‡ Supplying per kg diet: 18.9 mg retinol (vit A), 0.15 mg cholecalciferol (vit D3), 1038 mg α-tocopherol (vit E), 31.5 mg vitamin K, 31.5 mg vitamin B1, 31.5 mg vitamin B2, 157.5 mg D-pantothenic acid (vit B5), 315 mg niacin (vit B3), 0.79 mg biotin (vit B7), 0.315 mg vitamin B12, 47.3 mg vit B6, 1260 mg Fe, 225 mg Cu, 630 mg Mn (VA Vit SL/US Anti, Vilomix, Mørke, Denmark).
