# Supplementary material for: Postprandial PYY increase by resistant starch supplementation is independent of net portal appearance of short-chain fatty acids in pigs
Source: PLoS One. 2017 Oct 5;12(10):e0185927. doi: 10.1371/journal.pone.0185927 (PMC5628905; doi:10.1371/journal.pone.0185927)
Supplement: S2 Table — (DOCX) [file pone.0185927.s006.docx]

**Online Supporting Material**

**Supplemental Table 2:** Ingredients list of experimental diets used in experiment 2.

|  | **Experiment 2** | | |  |  |
| --- | --- | --- | --- | --- | --- |
|  | WWG | WAF | RAF | |  |
| Chemical composition (g/kg, as-fed basis) |  |  |  | |  |
| Whole-wheat grain | 813 | - | - | |  |
| Wheat aleurone^¶^ | - | 214 | - | |  |
| Rye flour (3-3.5% ash)* | - | - | 365 | |  |
| Wheat starch^†^ | - | 516 | 359 | |  |
| Wheat gluten | 36 | 116 | 116 | |  |
| Rapeseed oil | 76 | 79 | 85 | |  |
| Sugar | 15 | 15 | 15 | |  |
| Baker’s yeast | 15 | 15 | 15 | |  |
| Vitamin-mineral mixture^‡^ | 4 | 4 | 4 | |  |

WWG, whole-wheat grain; WAF, wheat aleurone flour; RAF, rye aleurone flour

* Raisio plc, Raisio, Finland

¶ ASP02, Bühler AG, Uzwil, Switzerland

† LCH A/S, Peter Bangs Vej 33, Frederiksberg, Denmark

‡ Supplying per kg diet: retinol, 660 µg; cholecalciferol, 12.5 µg; α-tocopherol, 30 mg; menadione, 11 mg; thiamin, 1 mg; riboflavin, 2 mg; D-pantothenic acid, 5·5 mg; niacin, 11 mg (available); biotin, 27.5 µg; cyanocobalamin, 11 µg; pyridoxine, 1.65 mg; Fe, 25 mg; Cu, 10 mg; Zn, 40 mg; Mn, 13.9 mg; Co, 0.15 mg; iodine, 0.01 mg; Se, 0.15 mg; and maize Ca_2_(PO_4_)_3_, K_2_PO_3_, NaCl and CaCO_3_ as a carrier (Solivit Mikro 106, Løvens Kemiske Fabrik, Vejen, Denmark
