# Supplementary material for: Postprandial PYY increase by resistant starch supplementation is independent of net portal appearance of short-chain fatty acids in pigs
Source: PLoS One. 2017 Oct 5;12(10):e0185927. doi: 10.1371/journal.pone.0185927 (PMC5628905; doi:10.1371/journal.pone.0185927)
Supplement: S3 Table — Correlations between net portal appearance of PYY (nmol/h) and total or individual short-chain fatty acids (SCFA; mmol/h) in experiments 1 and 2. (DOCX) [file pone.0185927.s007.docx]

**Online Supporting Material**

**Supplemental Table 3**: Correlations between net portal appearance of PYY (nmol/h) and total or individual short-chain fatty acids (SCFA; mmol/h) in experiments 1 and 2.

|  | | **Experiment 1** | | **Experiment 2** | | | |
| --- | --- | --- | --- | --- | --- | --- | --- |
|  | |  | | *First meal* | | *Second meal* | |
| Variables  (net portal appearance) | | Pearson correlation | *P*-value | Pearson correlation | *P*-value | Pearson correlation | *P*-value |
| PYY | Total SCFA | 0.1996 | 0.0952 | 0.2492 | 0.1486 | -0.0175 | 0.9219 |
|  | Acetate | 0.1835 | 0.1255 | 0.0304 | 0.0764 | 0.0349 | 0.8448 |
|  | Propionate | 0.1361 | 0.2614 | 0.0087 | 0.9613 | -0.0995 | 0.5756 |
|  | Butyrate | 0.1217 | 0.3120 | 0.3033 | 0.0765 | -0.0786 | 0.6587 |
